# Ability to detect depression in ethnic minority groups: a UK Biobank cohort study

**DOI:** 10.1136/bmjment-2026-302493

**Published:** 2026-07-13

**Authors:** Amy Ronaldson, Mel Ramasawmy, Paramjit S Gill, Rose Rickford, Hannah Frith, Andrea Martinez, Madiha Sajid, Khaula Ali, Lydia Poole

**Affiliations:** 1 Health Service and Population Research Department, King’s College London, London, UK; 2 Wolfson Institute of Population Health, Queen Mary University of London, London, UK; 3 Warwick Applied Health, University of Warwick, Coventry, UK; 4 School of Psychology, University of Surrey, Guildford, UK; 5 PPI Representative, London, UK; 6 College Road GP Practice, Woking, Surrey, UK; 7 Centre for Psychiatry and Mental Health, Wolfson Institute of Population Health, Queen Mary University of London, London, UK

**Keywords:** Depression

## Abstract

**Background:**

Evidence on ethnic differences in depression in the UK is mixed, and the suitability of current screening tools is under question.

**Objective:**

To examine ethnic differences in depression identification, symptom reporting and phenotypes among middle-aged and older adults in the UK.

**Methods:**

We assessed lifetime depression in 502 140 UK Biobank participants using an established algorithm. Depression symptoms were assessed using the Composite International Diagnostic Interview-Short Form data from the 2022 mental health follow-up. Latent class analysis (LCA) identified depression phenotypes. Logistic regression models adjusted for age, sex, deprivation and physical health assessed ethnic differences in depression identification, symptom reporting and phenotype membership.

**Findings:**

Overall, 23.7% of participants met study criteria for a lifetime history of depression. Compared with White participants, depression identification was significantly lower in all ethnic minority groups except the mixed group. Black, other Asian and South Asian participants were less likely to report core depression symptoms (prolonged sadness or loss of interest). LCA produced four phenotypes: major depression, somatic depression, non-somatic depression and subthreshold depression. Black (adjusted OR (aOR) 1.70, 95% CI 1.32 to 2.20), other Asian (aOR 1.73, 95% CI 1.21 to 2.46) and South Asian (aOR 1.63, 95% CI 1.26 to 2.10) participants were more likely than White participants to belong to the somatic depression phenotype.

**Conclusions:**

Lower rates of depression identification among ethnic minority groups may reflect limitations in how depression is defined, perceived and detected within these populations.

**Clinical implications:**

Depression may present more somatically in some ethnic minority groups. Clinicians may need to adopt more culturally informed assessment approaches to ensure accurate detection and appropriate support.

**WHAT IS ALREADY KNOWN ON THIS TOPIC**

- Evidence on ethnic differences in depression in the UK is limited, with most studies relying on self-report measures and tools that may not be culturally appropriate, leaving uncertainty about true prevalence and symptom patterns across ethnic groups.

**WHAT THIS STUDY ADDS**

- To our knowledge, this is the first population study analysing ethnic differences in depression identification, symptom endorsement and data-driven depression phenotypes using linked data to medical records to allow consideration of multiple clinical measures of depression alongside self-report. We show that lifetime depression is recorded less frequently in most ethnic minority groups, and the data indicate that Black, other Asian and South Asian adults with depression are more likely to present with somatic symptom profiles.

**HOW THIS STUDY MIGHT AFFECT RESEARCH, PRACTICE OR POLICY**

- These findings suggest that current screening and diagnostic approaches may under-detect depression or mischaracterise symptom presentations in some ethnic minority groups. Adopting more culturally informed assessment approaches and developing more inclusive measurement tools may help reduce inequities in recognition and care.

## Introduction

 Depression is one of the most common psychiatric illnesses globally.^1^ Prevalence is regionally patterned, with high estimates for Sub-Saharan Africa, North Africa and the Middle East, Australasia and high-income North America.^1^ There is evidence that depression symptom profiles differ across regions, suggesting that Western-based frameworks such as the Diagnostic and Statistical Manual of Mental Disorders (DSM) and International Classification of Diseases (ICD)^2^ may not be universally applicable.

While studies have examined mental health inequalities in the UK, comprehensive assessments focused on ethnic differences in depression prevalence and/or symptom profiles remain limited, and there is conflicting evidence. In the UK, some population studies indicate higher levels of depression among ethnic minority groups using structured questionnaire data focusing on recent/current symptoms,^3^ but an analysis of UK Biobank data indicates the opposite when using probable lifetime depression data.^4^ These studies have not considered heterogeneity in symptom profiles, which might account for variability in the predictive value of existing screening tools.^5^

Our recent scoping review of symptom heterogeneity for the South Asian diaspora revealed that commonly reported depressive symptoms include physical pain, heart-related symptoms and repetitive negative thinking, none of which are included in ICD-11 diagnostic criteria for depressive disorders.^6^

This study aims to examine ethnic differences in depression identified through multiple data sources and to explore heterogeneity in depressive symptoms across ethnic groups within the UK Biobank cohort. In addition, we examine ethnic differences in how depressive symptoms group together to assess the prevalence of different depression phenotypes in this cohort.

## Methods

### Study design and participants

This cross-sectional cohort study uses UK Biobank data from over 500 000 adults aged 40–69 years at recruitment between 2006 and 2010. The initial assessment took place at 22 assessment centres across England, Scotland and Wales,^7^ where sociodemographic, lifestyle and medical information was collected via questionnaire and computer-assisted interview. Hospital inpatient data (England: Hospital Episode Statistics (HES) Admitted Patient Care (APC), Wales: Patient Episode Database for Wales APC and Scotland: Scottish Morbidity Record (SMR01 and SMR04)) were available for ~90% participants and primary care records for ~45%. In 2016 and 2022, a subsample completed an online Mental Health Questionnaire (MHQ) assessing depression via self-report and validated measures, including the Composite International Diagnostic Interview-Short Form (CIDI-SF).^8^ UK Biobank is a volunteer cohort with known selection biases: participants tend to be healthier, more affluent and less ethnically diverse than the general UK population. Therefore, prevalence estimates from this study reflect patterns within the UK Biobank cohort and should not be interpreted as representative of the population.

This study was carried out as part of the Prescribing Antidepressants in Primary Care: Ethnic inequalities in tReatment (PAPER) study.^9^ A detailed protocol was preregistered on the Open Science Framework (https://doi.org/10.17605/OSF.IO/M7RJQ) in August 2025. All analyses and reporting were conducted in accordance with Strengthening the Reporting of Observational Studies in Epidemiology (STROBE) guidelines.^10^

### Exposure: ethnicity

Ethnicity was recorded at the baseline assessment. To maximise sample size in the current study, ethnicity was collapsed into the following groups based on the UK’s Office of National Statistics classifications: Black or Black British (African, Caribbean and any other Black background), mixed (White and Black Caribbean, White and Black African, White and Asian and any other mixed background), other, other Asian (any other Asian background and Chinese), South Asian (Bangladeshi, Indian and Pakistani), White (White British, White Irish and any other White background) and missing/unknown. The missing/unknown category is heterogeneous and should not be interpreted as a coherent ethnic category but is included for transparency.

### Outcome: depression identification

We assessed depression identification across the full UK Biobank cohort. Given the chronic or episodic nature of depression, we focused on lifetime depression (ie, depression ever recorded). Participants were classified as having a lifetime history of depression if they met one or more of the three identification criteria:

1. For participants who completed at least one MHQ, we used the CIDI-SF to identify probable lifetime major depressive disorder using established criteria^11^ (see online supplemental table S1).
2. For participants who did not complete the MHQ, lifetime depression was determined using an established algorithm^12^ combining self-report and linked hospital inpatient data (see online supplemental table S2). Participants were considered to have a depression history if they met two of the following criteria: self-reported help-seeking, self-reported depression, self-reported antidepressant usage, depression based on symptoms added to the assessment protocol in the last 2 years of recruitment or a hospital admission with a primary or secondary diagnosis of depression.
3. People with a primary care record of depression were considered to have a lifetime history of depression (read codes in online supplemental table S3).

We created a combined definition of lifetime depression, classifying participants as having a lifetime history if they met any of the three criteria (1–3). This definition was used to estimate overall prevalence. We also analysed each source separately (CIDI-SF, established algorithm, primary care records) to assess whether ethnic differences varied by detection method.

### Outcome: depressive symptoms

We examined depressive symptoms in participants who met criteria for lifetime depression and completed the 2022 MHQ follow-up. The 2022 MHQ was chosen, as it was the most recent assessment and included a larger number of respondents than 2016 (2022: n=169 523 and 2016: n=157 366).

We also examined ethnic differences in the proportion of missingness on each CIDI-SF item as a proxy measure to assess whether items were potentially less relevant or taboo for certain ethnic groups.

### Covariates

Age and sex were measured via self-report at the baseline assessment. Neighbourhood deprivation was measured using the Townsend deprivation indices^13^ derived from aggregated data on car ownership, household overcrowding, occupation and unemployment. Higher scores were indicative of higher deprivation, and scores were converted to quintiles to aid interpretation. Physical health status was determined through counting the number of physical long-term conditions (LTCs) reported by the participant at the baseline assessment. The LTCs included in this study were based on a previously developed list^14^ and are listed in online supplemental table S4. A categorical variable was created: no LTCs, one, two, three, four or more LTCs .

### Statistical analysis

Variables were summarised as means and SD and proportions. Ethnic differences in demographic variables were assessed using one-way analysis of variance and χ^2^ tests.

Lifetime depression identification was presented for each ethnic group and was stratified by age group, sex, deprivation and physical health status. Logistic regression models assessed ethnic differences in depression identification. We present unadjusted, age- and sex-adjusted and fully adjusted models (age, sex, neighbourhood deprivation and number of physical LTCs). We also explored ethnic differences in the way depression was determined (ie, CIDI-SF, established depression algorithm or primary care record).

In those who completed both the 2022 MHQ and met our criteria for a lifetime history of depression, we present the prevalence (%) of different depression symptoms (CIDI-SF) across ethnic groups and the proportion of missingness for each item. We used fully adjusted logistic regression models to examine ethnic differences in depression symptoms and proportions of missing data. In this same subsample, we used latent class analysis (LCA) to explore depression phenotypes. The optimal number of classes was determined based on the lowest Akaike Information Criterion (AIC) and the Bayesian-Schwarz Information Criterion (BIC). Once the number of latent classes had been identified, each participant was assigned to the class for which they had the largest posterior probability. The LCA was estimated using full information maximum likelihood, allowing participants with missing responses on CIDI-SF items to contribute all available information without exclusion. We used logistic regression to examine ethnic differences in each depression phenotype membership.

All analyses were performed using STATA V.18.0 (StataCorp, College Station, Texas, USA).

### Sensitivity analysis

We assessed the prevalence of individual depression symptoms and the proportion of missingness on each item among those who completed the CIDI-SF in the 2016 MHQ to validate findings from 2022. For analysis of individual CIDI-SF items and associated missingness, p-values were adjusted using the Benjamini-Hochberg procedure to decrease the false discovery rate.

### Role of the funding source

The funder of the study had no role in study design, data collection, data analysis, data interpretation or writing of the report.

## Results

### Sample characteristics

The study sample comprised 502 140 participants who completed the baseline UK Biobank assessment. Sample characteristics are presented in table 1. The sample had a mean age at baseline of 57.1 years (SD=8.1 years), and 54.4% were females. The majority (94.1%) of participants were White, followed by Black (1.6%), South Asian (1.6%), other (0.9%), other Asian (0.7%), mixed (0.6%) and missing/unknown (0.5%) ethnicities. Ethnic minority participants tended to be younger at the baseline assessment (p<0.001) and more were from areas with high deprivation (p<0.001). The majority of participants from ethnic minority groups were not born in the UK (71.0%–90.7%), with the exception of the mixed ethnic group (31.6%).

**Table 1:** Sample characteristics by ethnic group

	Overall n=502 140	White n=472 372 (94.1)	Black n=8048 (1.6)	Mixed n=2950 (0.6)	Other n=4552 (0.9)	Other Asian n=3428 (0.7)	South Asian n=8015 (1.6)	Missing/unknown n=2775 (0.5)
	Mean±SD or N (%)	Mean±SD or N (%)	Mean±SD or N (%)	Mean±SD or N (%)	Mean±SD or N (%)	Mean±SD or N (%)	Mean±SD or N (%)	Mean±SD or N (%)
Born outside of UK	44 816 (8.9)	24 023 (5.1)	5714 (71.0)	931 (31.6)	3668 (80.5)	3108 (90.7)	7007 (87.4)	365 (19.4)
Age at baseline assessment	57.1±8.1	57.3±8.0	52.4±8.1	52.3±8.1	53.1±8.0	53.3±8.1	53.9±8.4	56.8±8.3
Under 45 years	45 212 (9.0)	40 072 (8.5)	1486 (18.5)	596 (20.2)	815 (17.9)	590 (17.2)	1378 (17.2)	275 (9.9)
45–54 years	140 046 (27.9)	128 196 (27.1)	3645 (45.3)	1264 (42.8)	1830 (40.2)	1355 (39.5)	2924 (36.5)	832 (30.0)
55–64 years	208 964 (41.6)	200 023 (42.3)	1997 (24.8)	787 (26.7)	1453 (31.9)	1085 (31.6)	2574 (32.1)	1045 (37.7)
Over 65 years	107 918 (21.5)	104 081 (22.0)	920 (11.4)	303 (10.3)	454 (10.0)	398 (11.6)	1139 (14.2)	623 (22.4)
Sex								
Male	228 890 (45.6)	215 128 (45.5)	3404 (42.3)	1103 (37.4)	1959 (43.0)	1583 (46.2)	4292 (53.5)	1511 (54.4)
Female	273 160 (54.4)	257 244 (54.5)	4644 (57.7)	1847 (62.6)	2593 (57.0)	1845 (53.8)	3723 (46.5)	1264 (45.6)
Townsend deprivation								
1 (least)	101 276 (20.2)	98 883 (31.0)	227 (2.8)	326 (11.0)	357 (7.9)	404 (11.8)	766 (9.6)	313 (11.3)
2	100 550 (20.0)	97 863 (20.7)	368 (4.6)	343 (11.6)	394 (8.7)	422 (12.3)	779 (9.7)	381 (13.7)
3	99 128 (19.8)	95 447 (20.2)	651 (8.1)	446 (15.1)	474 (10.4)	457 (13.4)	1215 (15.2)	438 (15.8)
4	100 430 (20.0)	93 285 (19.8)	1675 (20.9)	647 (21.9)	990 (21.8)	936 (27.4)	2319 (29.0)	578 (20.9)
5 (most)	100 132 (20.0)	86 332 (18.3)	5107 (63.6)	1187 (40.2)	2321 (51.2)	1197 (35.0)	2927 (36.6)	1061 (38.3)
Physical long-term conditions								
None	200 204 (39.9)	187 635 (39.7)	3150 (39.1)	1296 (43.9)	1999 (43.9)	1618 (47.2)	3192 (39.8)	1314 (47.4)
One	167 513 (33.4)	157 971 (33.4)	2766 (34.4)	942 (31.9)	1445 (31.7)	1109 (32.3)	2495 (31.1)	785 (28.3)
Two	85 410 (17.0)	80 518 (17.0)	1395 (17.3)	482 (16.3)	703 (15.4)	480 (14.0)	1438 (17.9)	394 (14.2)
Three	32 956 (6.6)	31 063 (6.6)	517 (6.4)	155 (5.2)	285 (6.3)	160 (4.7)	593 (7.4)	183 (6.6)
Four or more	16 057 (3.2)	15 185 (3.2)	220 (2.7)	75 (2.5)	120 (2.6)	61 (1.8)	297 (3.7)	99 (3.6)
Lifetime depression	118 950 (23.7)	113 640 (24.1)	1219 (15.1)	866 (29.4)	945 (20.8)	434 (12.7)	1326 (16.5)	520 (18.7)
Source of depression measure*								
Composite International Diagnostic Interview-Short Form	45 758 (38.5)	44 380 (39.0)	286 (23.5)	313 (36.1)	243 (25.7)	123 (28.3)	244 (18.4)	169 (32.5)
Primary care	32 379 (27.2)	30 887 (27.2)	286 (23.5)	209 (24.1)	249 (26.3)	127 (29.3)	460 (34.7)	161 (31.0)
Depression algorithm	76 058 (63.9)	72 413 (63.7)	882 (72.4)	593 (68.5)	668 (70.7)	275 (63.4)	899 (67.8)	328 (63.1)

* There is overlap between sources of depression measure, meaning proportions will exceed 100%.

### Ethnic differences in depression identification

Overall, 23.7% of participants met study criteria for a lifetime history of depression (table 1). Relative to White participants (24.1%), identification of depression was lower across all ethnic minority groups, with the exception of individuals of mixed ethnicity (29.4%). The lowest levels of depression were observed in Black (15.1%) and other Asian (12.7%) participants. Across the sample, most cases were identified via the depression algorithm (self-report and HES data; 63.9%), followed by the CIDI-SF (38.5%) and primary care records (27.2%). Patterns of depression identification across ethnic groups are presented in table 1 and online supplemental table S5. Ethnic differences in lifetime depression identification by age, sex, levels of deprivation and physical health status are detailed in online supplemental table S6.

**Figure 1:**
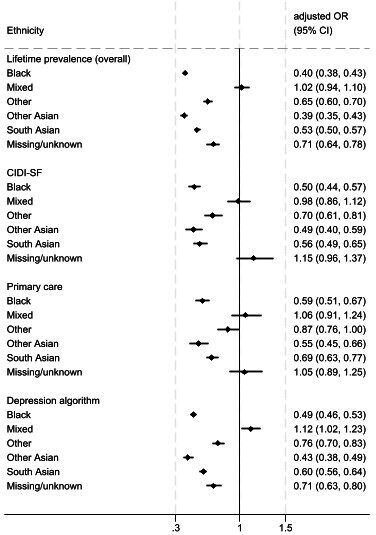
Fully adjusted OR and 95% CI for the association between ethnicity and lifetime depression prevalence (n=501 516), as well as depression measured by the CIDI-SF in those who completed one or both Mental Health Questionnaire follow-ups (n=218 344); depression recorded in primary care in those with primary care linkage available (n=229 590) and in those with depression detected using the depression algorithm at baseline (n=501 516). White ethnicity acts as the reference group. Models were adjusted for age, sex, Townsend deprivation index and number of physical long-term conditions. CIDI-SF, Composite International Diagnostic Interview-Short Form.

Figure 1 and online supplemental table S7 present results from logistic regression models examining ethnic differences in meeting study criteria for lifetime depression. Fully adjusted models indicate that all ethnic minority groups had significantly lower odds of being identified as having lifetime depression compared to the White group, except for participants of mixed ethnicity. Relative to White participants, the lowest odds were observed in Black (adjusted OR (aOR) 0.40, 95% CI 0.38 to 0.43) and other Asian individuals (aOR 0.39, 95% CI 0.35 to 0.43). Black, other, other Asian and South Asian groups were less likely than White participants to have depression detected on the CIDI-SF or in primary care compared with White participants. All ethnic minority groups were less likely to have depression detected using the algorithm at baseline, except for the mixed ethnic group, who were more likely than the White group to have depression detected by this method.

### Ethnic differences in depressive symptoms

Among those who completed the 2022 MHQ (n=175 241), 51 721 (29.5%) met study criteria for a lifetime history of depression, of whom 51.0% had depression detected by the CIDI-SF at this timepoint (online supplemental table S8). Logistic regression models (online supplemental table S9) revealed that Black, other Asian and South Asian participants with a history of depression were less likely to have depression detected by the CIDI-SF compared with White participants.

**Figure 2:**
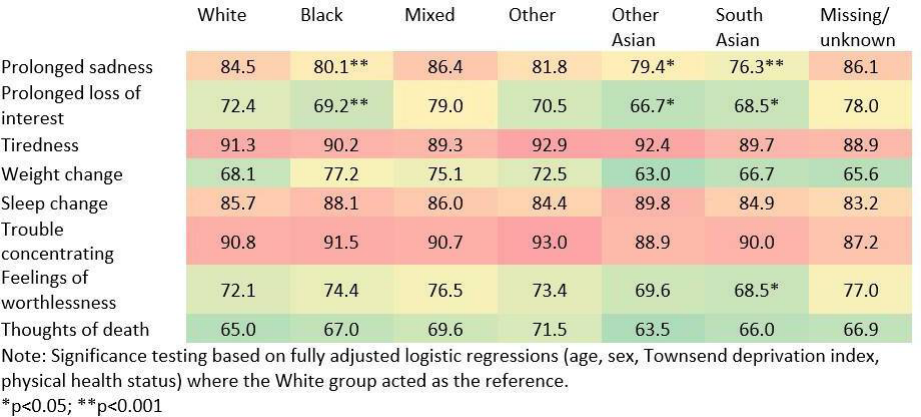
Heat map showing the percentage frequency of depression symptoms (Composite International Diagnostic Interview-Short Form) reported during the 2022 mental health questionnaire follow-up among individuals with a lifetime history of depression (n=51 721). Colour intensity ranges from green to red, with red indicating higher symptom frequency. Significance testing based on fully adjusted logistic regressions (age, sex. Townsend deprivation index, physical health status) where the White group acted as the reference. Significant differences detected using logistic regression models are indicated using asterisks; *p<0.05 and **p<0.001.

Figure 2 and online supplemental table S8 present symptoms from the CIDI-SF across ethnic groups. Fully adjusted logistic regression models (online supplemental table S9) showed that Black participants were significantly less likely than White participants to have experienced prolonged sadness (aOR 0.55, 95% CI 0.41 to 0.74) or prolonged loss of interest (anhedonia) (aOR 0.63, 95% CI 0.49 to 0.81)—the cardinal symptoms of depression as defined by DSM/ICD. Other Asian and South Asian participants were also less likely to experience these core symptoms when compared with White participants. South Asian participants were less likely to report feelings of worthlessness compared to White participants. The mixed ethnic group, the other ethnic group and those with missing/unknown ethnicity did not differ from the White group on any CIDI-SF item.

Relative to White participants, there were no ethnic differences in missingness on the core symptoms of depression (prolonged sadness and prolonged loss of interest), with the exception of the missing/unknown ethnic group, who had a significantly higher likelihood of missingness (online supplemental table S10). Black participants were more likely to have missing data on items relating to tiredness/low energy, weight change(s), feelings of worthlessness and thoughts of death. The other ethnic group had higher rates of missingness on items relating to tiredness/low energy and feelings of worthlessness. The other Asian group had a significantly higher likelihood of having missing data on items relating to tiredness/low energy, weight change and thoughts of death. South Asian participants had higher rates of missing data on all non-core depression items, relative to the White ethnic group.

### Depression phenotypes

We used LCA to determine depression phenotypes among those who completed both the 2022 MHQ and met our criteria for a lifetime history of depression (n=51 721). AIC and BIC values supported a four-class model (online supplemental table S11), which gave way to four distinct depression phenotypes (online supplemental figure S1 and online supplemental table S12):

1. Major depression: characterised by a high symptom burden across all domains (n=28 922, 55.9%).
2. Somatic depression subtype: characterised by low levels of core symptoms of depression (sadness and loss of interest) and high physical symptoms (n=12 133, 23.5%).
3. Non-somatic depression subtype: characterised by high levels of sadness with fewer somatic symptoms relative to other classes (n=7187, 13.9%).
4. Subthreshold depression: characterised by high levels of sadness but low levels of all other symptoms—could possibly represent situational distress (n=3479, 6.7%).

Ethnic differences in depression phenotypes are presented in table 2. Compared with White participants, those from Black, other Asian and South Asian groups were significantly less likely to belong to the major depression class. Conversely, participants from these ethnic groups were more likely to belong to the somatic depression class. There were no ethnic differences in non-somatic depression and subthreshold depression class subtypes.

**Table 2:** Ethnic differences in depression class membership among those with a lifetime history of depression who completed the Mental Health Follow-up Questionnaire in 2022 (n=51 721): proportions and fully adjusted multinomial regression models

	Overall (51 721)	White (50 223)	Black (300)	Mixed (332)	Other (266)	Other Asian (145)	South Asian (282)	Missing/unknown (173)
Major depression	28 922 (55.9)	28 084 (55.9)	166 (55.3)	216 (65.1)	148 (55.6)	71 (49.0)	138 (48.9)	99 (57.2)
Somatic depression subtype	12 133 (23.5)	11 742 (23.4)	86 (28.7)	63 (19.0)	70 (26.3)	46 (31.7)	85 (30.1)	41 (23.7)
Non-somatic depression subtype	7187 (13.9)	7016 (14.0)	31 (10.3)	31 (9.3)	31 (11.6)	20 (13.8)	39 (13.8)	19 (11.0)
Subthreshold depression	3479 (6.7)	3381 (6.7)	17 (5.7)	22 (6.6)	17 (6.4)	8 (5.5)	20 (7.1)	14 (8.1)
Fully adjusted logistic regression models examining ethnic differences in membership of each latent depression class								

	Major depression		Somatic depression subtype		Non-somatic depression		Subthreshold depression	
	aOR (95% CI)	P value	aOR (95% CI)	P value	aOR (95% CI)	P value	aOR (95% CI)	P value
White	Ref		Ref		Ref		Ref	
Black	**0.71 (0.56 to 0.90)**	0.004*	**1.70 (1.32 to 2.20)**	<0.001**	0.81 (0.55 to 1.17)	0.263	1.11 (0.67 to 1.81)	0.686
Mixed	1.13 (0.90 to 1.43)	0.279	0.95 (0.72 to 1.26)	0.747	0.69 (0.48 to 1.01)	0.055	1.26 (0.81 to 1.94)	0.306
Other	0.87 (0.68 to 1.12)	0.279	1.31 (1.00 to 1.73)	0.053	0.83 (0.57 to 1.22)	0.345	1.04 (0.64 to 1.71)	0.865
Other Asian	**0.67 (0.51 to 0.82)**	0.018*	**1.73 (1.21 to 2.46)**	0.002*	0.97 (0.59 to 1.57)	0.893	0.94 (0.46 to 1.93)	0.876
South Asian	**0.65 (0.51 to 0.82)**	<0.001**	**1.63 (1.26 to 2.10)**	<0.001**	1.00 (0.71 to 1.41)	0.980	1.21 (0.77 to 1.92)	0.404
Missing/unknown	1.08 (0.79 to 1.47)	0.628	1.02 (0.72 to 1.46)	0.892	0.73 (0.45 to 1.17)	0.192	1.21 (0.70 to 2.09)	0.503

Logistic regression models were adjusted for age, sex, Townsend deprivation index and number of physical long-term conditions.

*p<0.05 and **p<0.001.

aOR, adjusted OR.

### Sensitivity analyses

Among 2016 MHQ respondents (n=157 261), 48 464 (30.8%) met criteria for lifetime depression. CIDI-SF estimates are presented in online supplemental table S13, with item-level responses in online supplemental figure S2. Logistic regressions (online supplemental table S14) indicated that, as in 2022, Black and South Asian participants were less likely than White participants to report prolonged sadness or loss of interest. Unlike 2022, ethnic differences also appeared for non-core symptoms: Black participants reported less tiredness, while both Black and South Asian groups reported more weight change and less trouble concentrating. Patterns of item missingness (online supplemental table S15) were similar to 2022: no ethnic differences for core depression symptoms; higher missingness for South Asian participants on most non-core items, and, unlike 2022, no differences for Black participants, while the mixed ethnic group showed higher missingness on several non-core items.

After false discovery rate adjustment, some associations became non-significant in both 2022 and 2016 (eg, lower core symptoms in the other Asian group), but results for Black and South Asian participants remained largely consistent. In 2016, false discovery rate adjustment mainly reduced statistical significance rather than altering effect direction or magnitude.

## Discussion

In the UK Biobank sample of middle-aged and older adults, ethnic minority participants were less likely than White participants to be identified as having a lifetime history of depression, with the exception of the mixed ethnic group. This pattern predominantly held true across the three methods of detecting lifetime prevalence of depression, including the CIDI-SF depression screening tool, primary care records and algorithmic assessments, particularly for Black, other Asian and South Asian individuals.

When we examined individual depression symptoms using 2022 CIDI-SF responses (in those with a lifetime history of depression), we found significantly lower rates of prolonged sadness and anhedonia (core depression symptoms) among adults from Black, other Asian and South Asian groups. This finding was replicated in Black and South Asian groups using 2016 CIDI-SF data. Non-core depression items (eg, weight change and feelings of worthlessness) showed greater missingness in some ethnic minority groups, which might reflect limited relevance, increased stigma or interpretive ambiguity. Using data-driven methods, we derived four depression phenotypes: major depression, somatic depression, non-somatic depression and subthreshold depression. Participants from Black, other Asian and South Asian groups with a history of depression were less likely than White participants to be classified under the major depression phenotype and more likely to align with the somatic depression phenotype. This pattern may reflect lower endorsement of core depressive symptoms and higher rates of missing data on non-core items, potentially amplifying the representation of somatic features among some ethnic minority respondents.

Not all UK studies report lower depression prevalence in ethnic minority groups.^3 15^ Ahmad *et al* found weighted common mental health disorder prevalence of 16.2% in White British, 22.7% in Black, 16.3% in Asian and 19.3% in mixed/other participants.^3^ Williams *et al* reported depressive symptoms in 9.7% of White European, 15.5% of South Asian and 17.7% of Black Caribbean adults.^15^ Our comprehensive assessment of depression and linkage to hospital episode and primary care records increases confidence in our findings. Our findings are in line with the so-called ‘Black-White depression paradox’^16^ in which lower levels of depression have been observed among Black minority individuals despite increased exposure to social disadvantage. We suggest three different possible interpretations of the finding of lower depression identification among ethnic minorities. The first is that depression is less likely to be experienced by individuals from ethnic minority groups, hence the low rates of detection across different methods of assessment. The second is that the methods available to detect depression (medical records and screening instruments) are unable to accurately assess the true level of depression among ethnic minority groups, perhaps due to barriers in accessing medical settings or the unsuitability of screening tools which have typically been constructed using White populations in Western contexts^5^ and that fail to account for idioms of distress.^17^ The third is that ethnic minorities under-report symptoms, perhaps due to stigma.^18^

Given we observed ethnic differences in the patterning of symptoms and missingness on certain CIDI-SF items (particularly weight change, feelings of worthlessness and thoughts of death), our findings lend support to the second possibility and cast doubts over the suitability of existing methods of detecting depression for ethnic minority groups in the UK. While we are aware of work to validate screening tools such as the Patient Health Questionnaire-9 for use in ethnically diverse European populations,^19^ these tools are typically validated against DSM/ICD diagnostic criteria, which may not adequately capture ethnic minority experiences of depression.^6^ Given the lower identification of a lifetime history of depression also being observed from medical record data, our findings indicate that general practitioners may be underdiagnosing depression in ethnic minority patients.

The third possibility of under-reporting of symptoms may be supported by our finding of higher levels of somatic symptoms in ethnic minority groups. The somatisation hypothesis proposes that ethnic minority patients are more likely to present in clinical settings with physical rather than affective symptoms of distress as a more acceptable form of help-seeking and has been widely used to interpret the underdiagnosis of depression, although evidence to support this theory has been mixed.^20 21^ Stigma around mental illness is reportedly higher for ethnic minority groups than majority populations^22^ and has been shown to be detrimental to help-seeking behaviour.^23^ The increased stigma attached to psychological symptoms may particularly help explain our finding that the cardinal symptoms of depression (sadness and anhedonia) were less frequently endorsed by Black, other Asian and South Asian participants compared with White participants.

Financial deprivation has long been thought to predict a greater burden of depression,^24^ congruent with the social determinants model of health and illness. Our models adjusted for multiple markers of disadvantage through the inclusion of the Townsend deprivation index. While the highest levels of deprivation in our sample were observed in Black and South Asian participants, these groups also had lower levels of identified lifetime history of depression compared with White participants. These findings are consistent with data using US samples examining socioeconomic position and depression in African Americans, highlighting that low educational attainment may be a better predictor of depression for this group than household income.^25^ Given that education is not captured within the Townsend deprivation index, this should be explored in future studies.

Our findings also point to a disproportionately high depression burden for those of mixed ethnicity. A study using data from the UK Household Longitudinal Study showed that mixed ethnicity was associated with increased risk of mental health problems, and this was partly accounted for by higher levels of perceived personal discrimination in this group.^26^ Further work should explore this in greater depth, as it is likely that this group faces unique challenges relating to holding dual/multiple identities.

This study indicates that assessment tools and clinical decision-making are informed by clinical guidelines that may not fully capture the symptom experiences of depression for ethnic minority groups in the UK. Calls to revisit the underlying theory of depression require researchers to examine all elements that contribute to depression (risk factors, maintenance factors and outcomes) and their interactions^5^ for progress to be made in delivering culturally competent mental healthcare.

### Study strengths and limitations

The large overall sample size of the UK Biobank, plus the comprehensive baseline assessment, use of follow-up data and data linkages, allowed for a broad measure of depression using multiple methods of detection. Moreover, the use of data-driven techniques to determine depression symptom profiles provided a more detailed and nuanced understanding as opposed to simply assessing individual symptoms. However, the observational nature of the data introduces the possibility of residual confounding, which may affect the robustness of conclusions. Furthermore, our approach focused on ‘depression ever’, which did not allow us to account for the complex clinical course depression can take. There are also limitations of the UK Biobank dataset. UK Biobank participants are healthier, more affluent and less ethnically diverse than the general UK population, meaning the prevalence estimates produced in this study reflect patterns within the UK Biobank cohort only. These estimates should not be interpreted as representative of the UK population-level prevalence, as this selection profile may introduce selection bias and healthy volunteer bias. Furthermore, UK Biobank does not have sufficient data from ethnic minorities; the data are from middle-aged and older adults only, and the country of birth data indicate the majority of ethnic minority participants are first-generation immigrants; combined, these factors all pose issues for generalisability. There is a need to look at how well these findings are replicated in younger ethnic minority individuals born in the UK. Moreover, the marked imbalance in ethnic group sizes within the UK Biobank may have resulted in greater uncertainty for minority groups. These smaller sample sizes mean that estimates for some groups should be interpreted with appropriate caution. A further limitation, attributable to sample size constraints, was the reliance on broad ethnic categories, which mask meaningful within-group variation and limit the precision of ethnic comparisons, underscoring the need for more granular classifications in future work. Our combined lifetime depression definition drew on baseline assessment, linked primary care records and MHQ data, each of which is available for different subsets of participants. This uneven data availability meant that some individuals had more opportunities to be classified as having depression than others, which may have introduced selection bias and contributed to differences in case detection across groups.

## Conclusion

Our study has demonstrated lower rates of depression identification among ethnic minorities in the UK, coupled with a higher likelihood of a somatic symptom profile. This might reflect limitations in how depression is defined, perceived and detected in these populations. Our findings highlight the need for research to improve understanding of depression in ethnic minority populations, to advance methods for detecting depression and to address the cultural competency of existing diagnostic criteria.

## Supplementary material

10.1136/bmjment-2026-302493 online supplemental file 1

## Data Availability

Data may be obtained from a third party and are not publicly available.
