# Supplementary material for: Ability to detect depression in ethnic minority groups: a UK Biobank cohort study
Source: BMJ Ment Health. 2026 Jul 13;29(1):e302493. doi: 10.1136/bmjment-2026-302493 (PMC13365754; doi:10.1136/bmjment-2026-302493)
Supplement: online supplemental file 1 [file bmjment-29-1-s001.docx]

**Supplementary Material**

**The ability to detect depression in ethnic minority groups: A UK Biobank cohort study**

| Table S1. Defining lifetime prevalence of MDD using the CIDI-SF administered as part of the UK Biobank mental health follow-up questionnaires |
| --- |
| *A participant needed to meet the criteria described below to be considered as having ever had MDD. UK Biobank field IDs are provided alongside each symptom.* |
| At least one core symptom:  Persistent sadness (20446) = Yes OR Loss of interest (20441) = Yes  AND  How much of day (20436) = Most of day or All day long  AND  Did you feel this way (20439) = Almost every day or Every day  AND  Impairment (20440) = Somewhat or A lot  AND  Total number of symptoms endorsed (core and others) >= 5 from the following:  Persistent sadness (core) (20446); Loss of interest (core) (20441); Tired or low energy (20449); Gain or loss of weight (20536) = Gain, Loss or Gain and loss; Sleep change (20532); Trouble concentrating (20435); Feeling worthless (20450); Thinking about death (20437) |
| Note: UK Biobank data field codes are provided in parentheses throughout  CIDI-SF=Composite International Diagnostic Interview – short form; MDD = major depressive disorder |

| **Table S2.** Criteria for defining depression in UK Biobank participants who have not completed a mental health follow-up questionnaire[12]. To meet the criteria for depression, participants will need to have at least two of the measures below. | |
| --- | --- |
| 1. Help-seeking | A positive response to either of these questions asked at baseline:  ‘Have you ever seen a general practitioner for nerves, anxiety, tension or depression?’ (2090)  ‘Have you ever seen a psychiatrist for nerves, anxiety, tension or depression?’ (2100) |
| 2. Self-reported depression | Reporting having experienced depression (past or present) during the interview at baseline (20002) |
| 3. Antidepressant usage* | Reporting taking antidepressant medications during the verbal interview at baseline (20003) |
| 4. Depression (Smith et al[34]) | During the last two years of recruitment, questions on depressive symptoms were added to the assessment protocol (n=172,751). Items assessing depression were based on the PHQ-7. Three types of depression were defined:   - Single probable episode of major depression (20123) - Probably recurrent major depression (moderate) (20124) - Probable recurrent major depression (severe) (20125) |
| 5. Hospital (ICD-10) | Linked hospital inpatient admission: a primary (41202) or secondary (41204) International Classification of Diseases (ICD)-10 diagnosis of a depressive mood disorder from linked hospital admission records. |
| Note: UK Biobank data field codes are provided in parentheses throughout.  * In the current study, we included eight of the most commonly prescribed antidepressants in England between 2005 and 2015 (citalopram, duloxetine, fluoxetine, mirtazapine, paroxetine, sertraline, trazodone, venlafaxine)[35], as well as escitalopram. Amitriptyline and dosulepin were not included in this measure seeing as amitriptyline is often used to treat sleep problems and pain, and dosulepin is not recommended in the UK for the treatment of depression[36]  ICD=International Classification of Disease; PHQ=Patient Health Questionnaire | |

| **Table S3.** Primary care Read codes used to define depression | | | |
| --- | --- | --- | --- |
| **Readv2 code** | **Term v2** | **Readv3 code** | **Term v3** |
| E204. | neurotic_depression_reactive_type | XE1YC | reactive_depression |
| Eu323 | severe_depressive_episode_with_psychotic_symptoms | XE1ZZ | severe_depressive_episode_with_psychotic_symptoms |
| E1120 | single_major_depressive_episode_unspecified | E1120 | single_major_depressive_episode_unspecified |
| E1123 | single_major_depressive_episode_severe_without_mention_of_psychosis | E1123 | single_major_depressive_episode_severe_without_mention_of_psychosis |
| Eu329 | single_major_depressive_episode_severe_with_psychosis_psychosis_in_remission | XaX53 | Single major depress ep, severe with psych, psych in remission |
| E1124 | single_major_depressive_episode_severe_with_psychosis | E1124 | single_major_depressive_episode_severe_with_psychosis |
| E112z | single_major_depressive_episode_nos | E112z | single_major_depressive_episode_nos |
| E1122 | single_major_depressive_episode_moderate | E1122 | single_major_depressive_episode_moderate |
| E1121 | single_major_depressive_episode_mild | E1121 | single_major_depressive_episode_mild |
| E1125 | single_major_depressive_episode_in_partial_or_unspecified_remission | E1125 | single_major_depressive_episode_in_partial_or_unspecified_remission |
| E1126 | single_major_depressive_episode_in_full_remission | E1126 | single_major_depressive_episode_in_full_remission |
| Eu32. | depressive_episode | XE1Y0 | single_major_depressive_episode |
| E112. | single_major_depressive_episode | XE1Y0 | single_major_depressive_episode |
| Eu327 | major_depression_severe_without_psychotic_symptoms | XSGom | severe_major_depression_without_psychotic_features |
| Eu328 | major_depression_severe_with_psychotic_symptoms | XSGon | Severe major depression with psychotic features |
| Eu322 | severe_depressive_episode_without_psychotic_symptoms | XE1ZY | severe_depressive_episode_without_psychotic_symptoms |
| E118. | seasonal_affective_disorder | X761L | Seasonal affective disorder |
| E1130 | recurrent_major_depressive_episodes_unspecified | E1130 | recurrent_major_depressive_episodes_unspecified |
| E1133 | recurrent_major_depressive_episodes_severe_without_mention_of_psychosis | E1133 | recurrent_major_depressive_episodes_severe_without_mention_of_psychosis |
| Eu32A | recurrent_major_depressive_episodes_severe_with_psychosis_psychosis_in_remission | XaX54 | recurrent_major_depressive_episodes_severe_with_psychosis_psychosis_in_remission |
| E1134 | recurrent_major_depressive_episodes_severe_with_psychosis | E1134 | recurrent_major_depressive_episodes_severe_with_psychosis |
| E1132 | recurrent_major_depressive_episodes_moderate | E1132 | recurrent_major_depressive_episodes_moderate |
| E1131 | recurrent_major_depressive_episodes_mild | E1131 | recurrent_major_depressive_episodes_mild |
| E1135 | recurrent_major_depressive_episodes_in_partial_or_unspecified_remission | E1135 | recurrent_major_depressive_episodes_in_partial_or_unspecified_remission |
| E1136 | recurrent_major_depressive_episodes_in_full_remission | E1136 | recurrent_major_depressive_episodes_in_full_remission |
| E113. | recurrent_major_depressive_episode | XE1Y1 | recurrent_major_depressive_episodes |
| E113z | recurrent_major_depressive_episode_nos | E113z | recurrent_major_depressive_episode_nos |
| Eu33z | recurrent_depressive_disorder_unspecified | XE1Zf | recurrent_depressive_disorder_unspecified |
| Eu334 | recurrent_depressive_disorder_currently_in_remission | Eu334 | recurrent_depressive_disorder_currently_in_remission |
| Eu332 | recurrent_depressive_disorder_current_episode_severe_without_psychotic_symptoms | XE1Zd | recurrent_depressive_disorder_current_episode_severe_without_psychotic_symptoms |
| Eu333 | recurrent_depressive_disorder_current_episode_severe_with_psychotic_symptoms | XE1Ze | recurrent_depressive_disorder_current_episode_severe_with_psychotic_symptoms |
| Eu331 | recurrent_depressive_disorder_current_episode_moderate | Eu331 | recurrent_depressive_disorder_current_episode_moderate |
| Eu330 | recurrent_depressive_disorder_current_episode_mild | Eu330 | recurrent_depressive_disorder_current_episode_mild |
| Eu33. | recurrent_depressive_disorder | XE1Zc | recurrent_depressive_disorder |
| E1137 | recurrent_depression | E1137 | recurrent_depression |
| E291. | prolonged_depressive_reaction | E291. | prolonged_depressive_adjustment_reaction |
| Eu33y | other_recurrent_depressive_disorders | Eu33y | other_recurrent_depressive_disorders |
| Eu32y | other_depressive_episodes | XE1Za | other_depressive_episodes |
| Eu326 | major_depression_moderately_severe | XSGol | moderate_major_depression |
| Eu321 | moderate_depressive_episode | Eu321 | moderate_depressive_episode |
| Eu412 | mixed_anxiety_and_depressive_disorder | X00Sb | mixed_anxiety_and_depressive_disorder |
| Eu325 | major_depression_mild | XSGok | mild_major_depression |
| Eu320 | mild_depressive_episode | Eu320 | mild_depressive_episode |
| Eu324 | mild_depression | XaCIs | mild_depression |
| E11z2 | masked_depression | X00SU | masked_depression |
| Eu341 | dysthymia | E2112 | dysthymia |
| Eu32z | depressive_episode_unspecified | XE1Zb | depressive_episode_unspecified |
| E2B.. | depressive_disorder_nec | E2B.. | depressive_disorder_nec |
| E2B1. | chronic_depression | E2B1. | chronic_depression |
| E290z | brief_depressive_reaction_nos | E290z | brief_depressive_reaction_nos |
| E11y2 | atypical_depressive_disorder | E11y2 | atypical_depressive_disorder |
| Eu32B | antenatal_depression | XaY2C | antenatal_depression |
| E135. | agitated_depression | X00SQ | agitated_depression |
| E2003 | Anxiety with depression |  |  |
| Eu34 | Persistent mood affective disorders | X00SO | Depressive disorder |
| Eu3y1 | [X]Other recurrent mood affective disorders / [X]Recurrent brief depressive episodes | X00SR | Endogenous depression |
| X00SO | Depressive disorder | X00SS | Endogenous depression first episode |
| X00SR | Endogenous depression | Xa0wV | Recurrent brief depressive disorder |
| X00SS | Endogenous depression first episode | XaB9J | Depression NOS |
| Xa0wV | Recurrent brief depressive disorder | XaCHr | [X]Single episode agitated depression w'out psychotic symptoms |
| XaB9J | Depression NOS | XaCHs | [X]Single episode major depression w'out psychotic symptoms |
| XaCHr | [X]Single episode agitated depression w'out psychotic symptoms | XaCIu | Severe depression |
| XaCHs | [X]Single episode major depression w'out psychotic symptoms | XM1GC | Endogenous depression - recurrent |
| XaCIu | Severe depression | XSEGJ | Major depressive disorder |
| XM1GC | Endogenous depression - recurrent |  |  |
| XSEGJ | Major depressive disorder |  |  |

| **Table S4.** List of physical long-term conditions |
| --- |
| Anaemia  Asthma  Atrial fibrillation  Bronchiectasis  Cancer  Chronic fatigue syndrome  Chronic kidney disease  Chronic obstructive pulmonary disorder  Chronic sinusitis  Coronary heart disease  Connective tissue disorders (e.g. systemic lupus erythematosis; polymyositis)  Dementia  Diabetes  Diverticular disease  Dyspepsia  Endometriosis  Epilepsy  Eczema  Glaucoma  Heart failure  Hepatitis  Hypertension  Inflammatory bowel disorder  Irritable bowel syndrome  Liver disease  Méniere’s disease  Multiple sclerosis  Osteoporosis  Painful conditions (e.g. Sciatica; fibromyalgia)  Parkinson’s disease  Peripheral vascular disease  Polycystic ovarian syndrome  Prostate conditions  Psoriasis  Stroke  Thyroid conditions (e.g. Grave’s disease; thyroiditis) |

| **Table S5.** Lifetime prevalence of depression | | | | | | | | |
| --- | --- | --- | --- | --- | --- | --- | --- | --- |
|  | **Overall sample** | **Black** | **Mixed** | **Other** | **Other Asian** | **South Asian** | **White** | **Missing/Unknown** |
|  | *N=502,140* | *N=8048* | *N=2950* | *N=4522* | *N=3428* | *N=8015* | *N=472,372* | *N=2775* |
| **Lifetime depression** | 118,950 (23.7) | 1219 (15.1) | 866 (29.4) | 945 (20.8) | 434 (12.7) | 1326 (16.5) | 113,640 (24.1) | 520 (18.7) |
| **Source of depression measure** |  |  |  |  |  |  |  |  |
| *CIDI-SF* | 45,758 (38.5) | 286 (23.5) | 313 (36.1) | 243 (25.7) | 123 (28.3) | 244 (18.4) | 44,380 (39.0) | 169 (32.5) |
| *Primary care* | 32,379 (27.2) | 286 (23.5) | 209 (24.1) | 249 (26.3) | 127 (29.3) | 460 (34.7) | 30,887 (27.2) | 161 (31.0) |
| *Depression algorithm* | 76,058 (63.9) | 882 (72.4) | 593 (68.5) | 668 (70.7) | 275 (63.4) | 899 (67.8) | 72,413 (63.7) | 328 (63.1) |
| **Algorithm source** | *N=76058* | *N=882* | *N=593* | *N=668* | *N=275* | *N=899* | *N=72,413* | *N=328* |
| *Help-seeking* | 75,524 (99.3) | 873 (99.0) | 587 (99.0) | 652 (97.6) | 272 (98.9) | 879 (97.8) | 71,795 (99.4) | 286 (87.2) |
| *Self-reported depression* | 29,490 (38.8) | 254 (28.8) | 226 (38.1) | 237 (35.5) | 93 (33.8) | 311 (34.6) | 28,215 (39.0) | 154 (47.0) |
| *Self-reported antidepressant* | 29,453 (38.7) | 234 (26.5) | 198 (33.4) | 226 (33.8) | 79 (28.7) | 290 (32.3) | 28,294 (39.1) | 132 (40.2) |
| *Depression (Smith et al)* | 32,608 (42.9) | 558 (63.3) | 286 (48.2) | 315 (47.2) | 159 (57.8) | 503 (56.0) | 30,671 (42.4) | 116 (35.4) |
| *Depression HES* | 28,129 (37.0) | 299 (33.9) | 208 (35.1) | 251 (37.6) | 88 (32.0) | 310 (34.5) | 26,838 (37.1) | 135 (41.2) |
| **Algorithm count** | *N=76058* | *N=882* | *N=593* | *N=668* | *N=275* | *N=899* | *N=72,413* | *N=328* |
| *Two measures* | 46,231 (60.8) | 576 (65.3) | 379 (63.9) | 424 (63.5) | 179 (65.1) | 577 (64.2) | 43,890 (60.6) | 206 (62.8) |
| *Three measures* | 18,378 (24.2) | 190 (21.5) | 121 (20.4) | 155 (23.2) | 57 (20.7) | 179 (19.9) | 17,597 (24.3) | 79 (24.1) |
| *Four measures* | 9637 (12.7) | 84 (9.5) | 81 (13.7) | 77 (11.5) | 33 (12.0) | 113 (12.6) | 9208 (12.7) | 41 (12.5) |
| *Five measures* | 1812 (2.4) | 32 (3.6) | 12 (2.0) | 12 (1.8) | 6 (2.2) | 30 (3.3) | 1718 (2.4) | 2 (0.6) |
| CIDI-SF=Composite International Diagnostic Interview – short form; HES=Hospital Episode Statistics  Note: Categories are not mutually exclusive. Participants may meet criteria for depression in more than one algorithm source, so percentages sum to more than 100%. | | | | | | | | |

| **Table S6.** Lifetime prevalence of depression across age group, gender, deprivation, and physical long-term conditions | | | | | | | | |
| --- | --- | --- | --- | --- | --- | --- | --- | --- |
|  | **Overall sample** | **Black** | **Mixed** | **Other** | **Other Asian** | **South Asian** | **White** | **Missing/**  **Unknown** |
|  | *N=502,140* | *N=8048* | *N=2950* | *N=4522* | *N=3428* | *N=8015* | *N=472,372* | *N=2775* |
|  | *N(%)* | *N(%)* | *N(%)* | *N(%)* | *N(%)* | *N(%)* | *N(%)* | *N(%)* |
| **Lifetime depression** | 118,950 (23.7) | 1219 (15.1) | 866 (29.4) | 945 (20.8) | 434 (12.7) | 1326 (16.5) | 113,640 (24.1) | 520 (18.7) |
| **Age group** |  |  |  |  |  |  |  |  |
| *Under 45y* | 12334 (27.3) | 253 (17.0) | 183 (30.7) | 164 (20.1) | 85 (14.4) | 253 (18.4) | 11,385 (28.4) | 49 (17.8) |
| *45 to 54y* | 38474 (27.5) | 600 (16.5) | 408 (32.3) | 406 (22.2) | 182 (13.4) | 517 (17.7) | 36,246 (28.3) | 207 (24.9) |
| *55 to 64y* | 48807 (23.4) | 291 (14.6) | 217 (27.6) | 296 (20.4) | 132 (12.2) | 418 (16.2) | 47,347 (23.7) | 193 (18.5) |
| *65y and older* | 19089 (17.7) | 75 (8.1) | 58 (19.1) | 79 (17.4) | 35 (8.8) | 137 (12.0) | 18,662 (17.9) | 71 (11.4) |
| **Gender** |  |  |  |  |  |  |  |  |
| *Male* | 40081 (17.5) | 330 (9.7) | 235 (21.3) | 335 (17.1) | 184 (11.6) | 559 (13.0) | 38,289 (17.8) | 243 (16.1) |
| *Female* | 78623 (28.8) | 889 (19.1) | 631 (34.2) | 610 (23.5) | 250 (13.5) | 767 (20.6) | 75,351 (29.3) | 277 (21.9) |
| **Deprivation** |  |  |  |  |  |  |  |  |
| *1* | 20651 (20.4) | 35 (15.4) | 87 (26.7) | 57 (16.0) | 41 (10.1) | 114 (14.9) | 20315 (20.5) | 56 (17.9) |
| *2* | 21940 (21.8) | 56 (15.2) | 73 (21.3) | 77 (19.5) | 31 (7.3) | 109 (14.0) | 21577 (22.0) | 72 (18.9) |
| *3* | 22994 (23.2) | 81 (12.4) | 128 (28.7) | 107 (22.6) | 63 (13.8) | 188 (15.5) | 22389 (23.5) | 85 (19.4) |
| *4* | 25224 (25.1) | 276 (16.5) | 173 (26.7) | 225 (22.7) | 107 (11.4) | 380 (16.4) | 24001 (25.7) | 110 (19.0) |
| *5* | 27708 (27.7) | 767 (15.0) | 404 (34.0) | 477 (20.5) | 190 (15.9) | 534 (18.2) | 25180 (29.2) | 197 (18.6) |
| **Physical LTCs** |  |  |  |  |  |  |  |  |
| *None* | 44458 (20.5) | 374 (11.9) | 331 (25.5) | 338 (16.9) | 175 (10.8) | 438 (13.7) | 39,140 (20.9) | 178 (13.5) |
| *1* | 38877 (23.5) | 410 (14.8) | 271 (28.9) | 280 (19.4) | 141 (12.7) | 411 (16.5) | 37,393 (23.7) | 165 (21.0) |
| *2* | 21170 (26.9) | 245 (17.6) | 174 (36.1) | 183 (26.0) | 68 (14.2) | 258 (17.9) | 21,678 (26.9) | 88 (22.3) |
| *3* | 8982 (31.2) | 112 (21.7) | 56 (36.1) | 82 (28.8) | 29 (18.1) | 120 (20.2) | 9538 (30.7) | 50 (27.3) |
| *4 or more* | 5217 (39.8) | 78 (35.4) | 34 (45.3) | 62 (51.7) | 21 (34.4) | 99 (33.3) | 5891 (38.8) | 39 (39.4) |
| LTC=long-term condition | | | | | | | | |

| **Table S7.** Logistic regression models assessing associations between ethnicity and lifetime depression prevalence | | | | |
| --- | --- | --- | --- | --- |
| Unadjusted logistic regression | | | | |
|  | Lifetime depression | CIDI-SF | Primary care | Algorithm |
|  | N=502,140 | N=218,593 | N=229,933 | N=502,140 |
|  | *OR (95% CI)* | *OR (95% CI)* | *OR (95% CI)* | *OR (95% CI)* |
| White | Ref | Ref | Ref | Ref |
| Black | 0.56 (0.53 to 0.60)** | 0.77 (0.68 to 0.88)** | 0.78 (0.69 to 0.88)** | 0.68 (0.63 to 0.73)** |
| Mixed | 1.31 (1.21 to 1.42)** | 1.38 (1.21 to 1.57)** | 1.33 (1.14 to 1.54)** | 1.39 (1.27 to 1.52)** |
| Other | 0.83 (0.77 to 0.89)** | 0.89 (0.77 to 1.02) | 1.05 (0.92 to 1.20) | 0.95 (0.87 to 1.03) |
| Other Asian | 0.46 (0.41 to 0.51)** | 0.59 (0.49 to 0.71)** | 0.62 (0.52 to 0.74)** | 0.48 (0.423 to 0.54)** |
| South Asian | 0.62 (0.59 to 0.66)** | 0.65 (0.57 to 0.75)** | 0.80 (0.73 to 0.89)** | 0.70 (0.65 to 0.75)** |
| Missing/unknown | 0.73 (0.66 to 0.80)** | 1.04 (0.88 to 1.24) | 1.06 (0.90 to 1.26) | 0.74 (0.66 to 0.83)** |
| Age and sex-adjusted logistic regression | | | | |
|  | Lifetime depression | CIDI-SF | Primary care | Algorithm |
|  | N=502,140 | N=218,593 | N=229,933 |  |
|  | *OR (95% CI)* | *OR (95% CI)* | *OR (95% CI)* | *OR (95% CI)* |
| White | Ref | Ref | Ref | Ref |
| Black | 0.48 (0.45 to 0.51)** | 0.59 (0.51 to 0.67)** | 0.70 (0.62 to 0.79)** | 0.63 (0.59 to 0.68)** |
| Mixed | 1.11 (1.02 to 1.20)* | 1.05 (0.92 to 1.20) | 1.16 (0.99 to 1.35) | 1.26 (1.15 to 1.38)** |
| Other | 0.73 (0.68 to 0.79)** | 0.76 (0.66 to 0.88)** | 0.97 (0.85 to 1.11) | 0.89 (0.82 to 0.97)* |
| Other Asian | 0.41 (0.37 to 0.45)** | 0.50 (0.41 to 0.61)** | 0.58 (0.48 to 0.70)** | 0.46 (0.41 to 0.52)** |
| South Asian | 0.60 (0.56 to 0.63)** | 0.59 (0.51 to 0.68)** | 0.78 (0.71 to 0.86)** | 0.70 (0.65 to 0.75)** |
| Missing/unknown | 0.75 (0.68 to 0.83)** | 1.18 (0.99 to 1.41) | 1.10 (0.93 to 1.30) | 0.77 (0.68 to 0.86)** |
| Fully adjusted logistic regression (N=501,516) | | | | |
|  | Lifetime depression | CIDI-SF | Primary care | Algorithm |
|  | N=501,516 | N=218,344 | N=229,590 | N=501,516 |
|  | *OR (95% CI)* | *OR (95% CI)* | *OR (95% CI)* | *OR (95% CI)* |
| White | Ref | Ref | Ref | Ref |
| Black | 0.40 (0.38 to 0.43)** | 0.50 (0.44 to 0.57)** | 0.59 (0.51 to 0.67)** | 0.49 (0.46 to 0.53)** |
| Mixed | 1.02 (0.94 to 1.10) | 0.98 (0.86 to 1.12) | 1.06 (0.91 to 1.24) | 1.12 (1.02 to 1.23)* |
| Other | 0.65 (0.60 to 0.70)** | 0.70 (0.61 to 0.81)** | 0.87 (0.76 to 1.00) | 0.76 (0.70 to 0.83)** |
| Other Asian | 0.39 (0.35 to 0.43)** | 0.49 (0.40 to 0.59)** | 0.55 (0.45 to 0.66)** | 0.43 (0.38 to 0.49)** |
| South Asian | 0.53 (0.50 to 0.57)** | 0.56 (0.49 to 0.65)** | 0.69 (0.63 to 0.77)** | 0.60 (0.56 to 0.64)** |
| Missing/unknown | 0.71 (0.64 to 0.78)** | 1.15 (0.96 to 1.37) | 1.05 (0.89 to 1.25) | 0.71 (0.63 to 0.80)** |
| CIDI-SF depression was assessed in those who completed one or both Mental Health Questionnaires. Primary care depression diagnoses were assessed in those who had primary care linkage available.  *p<0.05, **p<0.001  CI=confidence interval; CIDI-SF=Composite International Diagnostic Interview – short form; OR=odds ratio | | | | |

| **Table S8.** Ethnic difference in CIDI-SF scores and items among those who completed the MHQ follow-up in 2022 with a lifetime history of depression (N=51,721) | | | | | | | | | |
| --- | --- | --- | --- | --- | --- | --- | --- | --- | --- |
|  | CIDI-SF Depression | CIDI-SF core symptom: Prolonged sadness | CIDI-SF core symptom: Prolonged loss of interest | CIDI-SF non-core symptom:  Felt tired or had little energy | CIDI-SF non-core symptom:  Significant weight change | CIDI-SF non-core symptom:  Trouble sleeping (too much or too little) | CIDI-SF non-core symptom:  Trouble concentrating | CIDI-SF non-core symptom:  Feelings of worthlessness | CIDI-SF non-core symptom:  Thoughts of death |
|  | N (%) | N (%) | N (%) | N (%) | N (%) | N (%) | N (%) | N (%) | N (%) |
| Overall sample  N=51721 | 26401 (51.0) | 42886 (84.4) | 36733 (72.4) | 37189 (91.3) | 26645 (68.2) | 33708 (85.7) | 36749 (90.8) | 29673 (72.1) | 26740 (65.1) |
| *Missing* |  | 896 (1.7) | 987 (1.9) | 10,991 (21.2) | 12,669 (24.5) | 12,376 (23.9) | 11,259 (21.8) | 10,590 (20.5) | 10,654 (20.6) |
| White  N=50223 | 25663 (51.0) | 41698 (84.5) | 35686 (72.4) | 36153 (91.3) | 25857 (68.1) | 32735 (85.7) | 35701 (90.8) | 28821 (72.1) | 25961 (65.0) |
| *Missing* |  | 852 (1.7) | 938 (1.9) | 10,639 (21.2) | 12,275 (24.4) | 12,010 (23.9) | 10,918 (21.7) | 10,252 (20.4) | 10,303 (20.5) |
| Black  N=300 | 154 (51.3) | 233 (80.1) | 200 (69.2) | 203 (90.2) | 169 (77.2) | 199 (88.1) | 216 (91.5) | 166 (74.4) | 152 (67.0) |
| *Missing* |  | 9 (3.0) | 11 (3.7) | 75 (25.0) | 81 (27.0) | 74 (24.7) | 64 (21.3) | 77 (25.7) | 73 (24.3) |
| Mixed  N=332 | 190 (57.2) | 280 (86.4) | 256 (79.0) | 243 (89.3) | 196 (75.1) | 221 (86.0) | 245 (90.7) | 205 (76.5) | 188 (69.6) |
| *Missing* |  | 8 (2.4) | 8 (2.4) | 60 (18.1) | 71 (21.4) | 75 (22.6) | 62 (18.7) | 64 (19.3) | 62 (18.7) |
| Other  N=266 | 141 (53.0) | 212 (81.8) | 182 (70.5) | 182 (92.9) | 148 (72.5) | 173 (84.4) | 186 (93.0) | 149 (73.4) | 148 (71.5) |
| *Missing* |  | 7 (2.6) | 8 (3.0) | 70 (26.3) | 62 (23.3) | 61 (22.9) | 66 (24.8) | 63 (23.7) | 59 (22.2) |
| Other Asian  N=145 | 66 (45.5) | 112 (79.4) | 94 (66.7) | 97 (92.4) | 63 (63.0) | 97 (89.8) | 96 (88.9) | 80 (69.6) | 68 (63.5) |
| *Missing* |  | 4 (2.8) | 2 (2.8) | 40 (27.8) | 45 (31.0) | 37 (25.5) | 37 (25.5) | 30 (20.7) | 38 (26.2) |
| South Asian  N=282 | 128 (45.4) | 209 (76.3) | 187 (68.5) | 183 (89.7) | 130 (66.7) | 174 (84.9) | 189 (90.0) | 148 (68.5) | 134 (66.0) |
| *Missing* |  | 8 (2.8) | 9 (3.2) | 78 (27.7) | 87 (30.8) | 77 (27.6) | 72 (25.5) | 66 (23.4) | 79 (28.0) |
| Missing/unknown  N=173 | 89 (51.4) | 142 (86.1) | 128 (78.0) | 128 (88.9) | 82 (65.6) | 109 (83.2) | 116 (87.2) | 104 (77.0) | 89 (66.9) |
| *Missing* |  | 8 (4.6) | 9 (5.2) | 29 (16.8) | 48 (27.7) | 42 (24.3) | 40 (23.1) | 38 (22.0) | 40 (23.1) |
| Note: Missing can mean not completed, ‘do not know’, or ‘prefer not to say’  CIDI-SF=Composite International Diagnostic Interview – short form | | | | | | | | | |

| **Table S9.** Ethnic difference in CIDI-SF scores and items among those who completed the MHQ follow-up in 2022 with a lifetime history of depression (N=51,721) | | | | | | | | | |
| --- | --- | --- | --- | --- | --- | --- | --- | --- | --- |
|  | CIDI-SF Depression | CIDI-SF core symptom: Prolonged sadness | CIDI-SF core symptom: Prolonged loss of interest | CIDI-SF non-core symptom:  Felt tired or had little energy | CIDI-SF non-core symptom:  Significant weight change | CIDI-SF non-core symptom:  Trouble sleeping (too much or too little) | CIDI-SF non-core symptom:  Trouble concentrating | CIDI-SF non-core symptom:  Feelings of worthlessness | CIDI-SF non-core symptom:  Thoughts of death |
|  | *OR (95% CI),*  *p value* | *OR (95% CI),*  *p value* | *OR (95% CI),*  *p value* | *OR (95% CI),*  *p value* | *OR (95% CI),*  *p value* | *OR (95% CI),*  *p value* | *OR (95% CI),*  *p value* | *OR (95% CI),*  *p value* | *OR (95% CI),*  *p value* |
|  | 51,643 | 50,748 | 50,657 | 40,669 | 38,997 | 39,286 | 40,402 | 41,067 | 41,002 |
| White | Ref | Ref | Ref | Ref | Ref | Ref | Ref | Ref | Ref |
| Black | 0.76 (0.60 to 0.96), 0.020* | 0.55 (0.41 to 0.74), <0.001** | 0.63 (0.49 to 0.81), <0.001** | 0.72 (0.46 to 1.12), 0.149 | 1.25 (0.91 to 1.73), 0.170 | 1.02 (0.68 to 1.54), 0.913 | 0.88 (0.55 to 1.39), 0.578 | 0.84 (0.62 to 1.14), 0.274 | 0.92 (0.70 to 1.21), 0.552 |
| Mixed | 1.00 (0.80 to 1.25), 0.966 | 0.92 (0.66 to 1.26), 0.575 | 1.10 (0.84 to 1.45), 0.483 | 0.68 (0.46 to 1.01), 0.057 | 1.19 (0.89 to 1.58), 0.238 | 0.88 (0.62 to 1.26), 0.494 | 0.83 (0.54 to 1.25), 0.370 | 1.00 (0.75 to 1.34), 0.984 | 1.11 (0.85 to 1.44), 0.441 |
| Other | 0.97 (0.76 to 1.24), 0.791 | 0.74 (0.54 to 1.02), 0.058 | 0.80 (0.61 to 1.05), 0.090 | 1.19 (0.69 to 2.06), 0.532 | 1.19 (0.87 to 1.63), 0.281 | 0.86 (0.59 to 1.26), 0.445 | 1.24 (0.72 to 2.15), 0.435 | 0.95 (0.69 to 1.30), 0.738 | 1.27 (0.94 to 1.72), 0.123 |
| Other Asian | 0.71 (0.51 to 0.99), 0.042* | 0.63 (0.42 to 0.95), 0.022* | 0.66 (0.46 to 0.94), 0.017* | 1.06 (0.52 to 2.20), 0.865 | 0.78 (0.52 to 1.19), 0.251 | 1.41 (0.75 to 2.65), 0.279 | 0.72 (0.39 to 1.32), 0.290 | 0.80 (0.53 to 1.20), 0.238 | 0.91 (0.61 to 1.35), 0.639 |
| South Asian | 0.67 (0.53 to 0.86), 0.001* | 0.52 (0.40 to 0.70), <0.001** | 0.71 (0.55 to 0.92), 0.006* | 0.77 (0.49 to 1.22), 0.265 | 0.87 (0.64 to 1.18), 0.379 | 0.86 (0.58 to 1.26), 0.435 | 0.80 (0.51 to 1.27), 0.349 | 0.69 (0.51 to 0.92), 0.012* | 0.98 (0.73 to 1.31), 0.879 |
| Missing/unknown | 1.02 (0.76 to 1.39), 0.876 | 1.20 (0.77 to 1.87), 0.534 | 1.41 (0.98 to 2.05), 0.119 | 0.78 (0.46 to 1.32), 0.361 | 0.96 (0.66 to 1.40), 0.822 | 0.85 (0.54 to 1.36), 0.501 | 0.69 (0.41 to 1.15), 0.156 | 1.29 (0.86 to 1.93), 0.222 | 1.09 (0.76 to 1.57), 0.627 |
| *p<0.05; **p<0.001  Fully adjusted analyses: Age, sex, Townsend deprivation quintile, number of physical long term conditions  CI=confidence interval; CIDI-SF=Composite International Diagnostic Interview – short form; OR=odds ratio  Note: Benjamini–Hochberg false discovery rate (5%) applied as a sensitivity analysis; contrasts with p‑values below 0.004 were considered significant after adjustment. | | | | | | | | | |

| **Table S10.** Ethnic difference in the likelihood of missingness for each CIDI-SF item among those who completed the MHQ follow-up in 2022 with a lifetime history of depression (N=51,721) | | | | | | | | |
| --- | --- | --- | --- | --- | --- | --- | --- | --- |
|  | CIDI-SF core symptom: Prolonged sadness | CIDI-SF core symptom: Prolonged loss of interest | CIDI-SF non-core symptom:  Felt tired or had little energy | CIDI-SF non-core symptom:  Significant weight change | CIDI-SF non-core symptom:  Trouble sleeping (too much or too little) | CIDI-SF non-core symptom:  Trouble concentrating | CIDI-SF non-core symptom:  Feelings of worthlessness | CIDI-SF non-core symptom:  Thoughts of death |
|  | *OR (95% CI),*  *p value* | *OR (95% CI),*  *p value* | *OR (95% CI),*  *p value* | *OR (95% CI),*  *p value* | *OR (95% CI),*  *p value* | *OR (95% CI),*  *p value* | *OR (95% CI),*  *p value* | *OR (95% CI),*  *p value* |
| White | Ref | Ref | Ref | Ref | Ref | Ref | Ref | Ref |
| Black | 1.64 (0.81 to 3.34), 0.171 | 1.88 (0.99 to 3.56), 0.052 | 1.53 (1.17 to 2.00), 0.002* | 1.36 (1.05 to 1.77), 0.020* | 1.31 (1.00 to 1.71), 0.051 | 1.24 (0.93 to 1.65), 0.134 | 1.73 (1.32 to 2.26), <0.001** | 1.54 (1.18 to 2.02), 0.002* |
| Mixed | 1.51 (0.74 to 3.05), 0.256 | 1.38 (0.68 to 2.79), 0.375 | 1.00 (0.75 to 1.33), 1.000 | 0.99 (0.76 to 1.29), 0.941 | 1.13 (0.87 to 1.47), 0.349 | 1.04 (0.79 to 1.38), 0.767 | 1.17 (0.89 to 1.54), 0.270 | 1.08 (0.82 to 1.43), 0.593 |
| Other | 1.54 (0.72 to 3.27), 0.264 | 1.60 (0.79 to 3.26), 0.191 | 1.45 (1.10 to 1.91), 0.009* | 1.00 (0.75 to 1.34), 0.975 | 1.04 (0.78 to 1.39), 0.784 | 1.32 (0.99 to 1.75), 0.054 | 1.35 (1.01 to 1.80), 0.040* | 1.21 (0.91 to 1.63), 0.194 |
| Other Asian | 1.68 (0.62 to 4.54), 0.310 | 1.53 (0.57 to 4.16), 0.400 | 1.58 (1.09 to 2.28), 0.015* | 1.52 (1.07 to 2.17), 0.021* | 1.23 (0.84 to 1.79), 0.282 | 1.41 (0.96 to 2.05), 0.076 | 1.15 (0.77 to 1.72), 0.500 | 1.53 (1.05 to 2.22), 0.025* |
| South Asian | 1.76 (0.87 to 3.57), 0.118 | 1.81 (0.93 to 3.54), 0.081 | 1.59 (1.22 to 2.07), 0.001* | 1.50 (1.16 to 1.94), 0.002* | 1.36 (1.05 to 1.78), 0.022* | 1.43 (1.09 to 1.88),0.010* | 1.37 (1.03 to 1.81), 0.028* | 1.70 (1.30 to 2.21), <0.001** |
| Missing/unknown | 2.72 (1.33 to 5.56), 0.006* | 2.82 (1.43 to 5.53), 0.003* | 0.74 (0.49 to 1.10), 0.137 | 1.17 (0.84 to 1.64), 0.358 | 1.02 (0.92 to 1.44), 0.925 | 1.08 (0.76 to 1.55), 0.666 | 1.10 (0.76 to 1.58), 0.613 | 1.16 (0.81 to 1.66), 0.412 |
| *p<0.05; **p<0.001  Fully adjusted analyses: Age, sex, Townsend deprivation quintile, number of physical long-term conditions  CI=confidence interval; CIDI-SF=Composite International Diagnostic Interview – short form; OR=odds ratio  Note: Benjamini–Hochberg false discovery rate (5%) applied as a sensitivity analysis; contrasts with p‑values below 0.011 were considered significant after adjustment. | | | | | | | | |

| **Table S11.** Latent class analysis: AIC and BIC values | | |
| --- | --- | --- |
| Number of classes | Akaike Information Criterion (AIC) | Bayesian-Schwarz Information Criterion (BIC) |
| 2 | 311442.7 | 311593.0 |
| 3 | 309271.4 | 309501.1 |
| 4 | 308604.3 | 308904.8 |
| 5 | Convergence not achieved | |

| **Table S12.** Proportion of endorsed CIDI-SF depression items in each latent depression class (N=51,721) | | | | | | | | | |
| --- | --- | --- | --- | --- | --- | --- | --- | --- | --- |
|  | **N** | **Prolonged sadness** | **Prolonged loss of interest** | **Tiredness** | **Weight change** | **Sleep change** | **Trouble concentrating** | **Worthlessness** | **Thoughts of death** |
| **Major depression** | 28922 (55.9) | 28901 (100.0) | 28834 (100.0) | 26187 (96.3) | 22229 (87.7) | 24555 (93.8) | 26830 (97.2) | 22222 (82.0) | 20149 (75.1) |
| **Somatic depression subtype** | 12133 (23.5) | 3708 (32.9) | 608 (5.4) | 3681 (95.0) | 2921 (81.0) | 3718 (99.0) | 3577 (94.7) | 1943 (49.1) | 2547 (63.1) |
| **Non-somatic depression subtype** | 7187 (13.9) | 6919 (96.3) | 5981 (83.5) | 6038 (91.4) | 334 (4.8) | 4214 (65.5) | 6143 (93.7) | 4564 (67.2) | 2935 (42.7) |
| **Subthreshold depression** | 3479 (6.7) | 3358 (96.6) | 1310 (37.8) | 1283 (41.8) | 1161 (37.2) | 1221 (41.0) | 649 (21.6) | 944 (28.7) | 1109 (33.4) |
| CIDI-SF=Composite International Diagnostic Interview – short form | | | | | | | | | |

| **Table S13.** Ethnic difference in CIDI-SF scores and items among those who completed the MHQ follow-up in 2016 with a lifetime history of depression (N=48,464) | | | | | | | | | |
| --- | --- | --- | --- | --- | --- | --- | --- | --- | --- |
|  | CIDI-SF Depression | CIDI-SF core symptom: Prolonged sadness | CIDI-SF core symptom: Prolonged loss of interest | CIDI-SF non-core symptom:  Felt tired or had little energy | CIDI-SF non-core symptom:  Significant weight change | CIDI-SF non-core symptom:  Trouble sleeping (too much or too little) | CIDI-SF non-core symptom:  Trouble concentrating | CIDI-SF non-core symptom:  Feelings of worthlessness | CIDI-SF non-core symptom:  Thoughts of death |
|  | N (%) | N (%) | N (%) | N (%) | N (%) | N (%) | N (%) | N (%) | N (%) |
| Overall sample  N=48,464 | 30758 (63.5) | 44,447 (91.8) | 38346 (79.3) | 39955 (93.4) | 27552 (70.1) | 37616 (90.9) | 39098 (92.5) | 30588 (71.7) | 28216 (66.1) |
| *Missing* |  | 60 (0.1) | 105 (0.2) | 5692 (11.7) | 9169 (18.9) | 7078 (14.6) | 6203 (12.8) | 5830 (12.0) | 5771 (11.9) |
| White  N=47,025 | 29845 (63.5) | 43173 (91.9) | 37213 (79.3) | 38807 (93.5) | 26667 (69.9) | 36491 (90.9) | 37985 (92.6) | 29691 (71.7) | 27349 (66.0) |
| *Missing* |  | 56 (0.1) | 101 (0.2) | 5504 (11.7) | 8886 (18.9) | 6873 (14.6) | 5994 (12.7) | 5626 (12.0) | 5583 (11.9) |
| Black  N=284 | 187 (65.8) | 251 (88.7) | 224 (79.2) | 222 (89.2) | 190 (82.6) | 233 (93.2) | 222 (89.2) | 163 (66.0) | 167 (67.6) |
| *Missing* |  | 1 (0.4) | 1 (0.4) | 35 (12.3) | 54 (19.0) | 34 (12.0) | 35 (12.3) | 37 (13.0) | 37 (13.0) |
| Mixed  N=324 | 200 (61.7) | 286 (88.8) | 255 (79.2) | 254 (92.7) | 203 (77.8) | 238 (89.1) | 252 (91.3) | 211 (76.2) | 195 (70.1) |
| *Missing* |  | 2 (0.6) | 2 (0.6) | 50 (15.4) | 63 (19.4) | 57 (17.6) | 48 (14.8) | 47 (14.5) | 46 (14.2) |
| Other  N=274 | 168 (61.3) | 246 (89.8) | 220 (80.3) | 217 (91.6) | 163 (76.5) | 216 (91.9) | 205 (89.1) | 170 (72.6) | 160 (65.8) |
| *Missing* |  | 0 (0.0) | 0 (0.0) | 37 (13.5) | 61 (22.3) | 39 (14.2) | 44 (16.1) | 40 (14.6) | 31 (11.3) |
| Other Asian  N=123 | 81 (65.8) | 111 (91.0) | 95 (77.2) | 101 (91.0) | 71 (66.4) | 106 (94.6) | 96 (89.7) | 73 (70.2) | 79 (71.8) |
| *Missing* |  | 1 (0.8) | 0 (0.0) | 12 (9.8) | 16 (13.0) | 11 (8.9) | 16 (13.0) | 19 (15.4) | 13 (10.6) |
| South Asian  N=269 | 162 (60.2) | 226 (84.0) | 201 (75.0) | 207 (91.2) | 165 (77.5) | 206 (90.3) | 201 (90.5) | 165 (72.7) | 160 (71.4) |
| *Missing* |  | 0 (0.0) | 1 (0.4) | 42 (15.6) | 56 (20.8) | 41 (15.2) | 47 (17.5) | 42 (15.6) | 45 (16.7) |
| Missing/unknown  N=165 | 115 (69.7) | 154 (93.3) | 138 (83.6) | 147 (96.1) | 93 (70.4) | 126 (88.7) | 137 (93.8) | 115 (78.8) | 106 (71.1) |
| *Missing* |  | 0 (0.0) | 0 (0.0) | 12 (7.3) | 33 (20.0) | 23 (13.9) | 19 (11.5) | 19 (11.5) | 16 (9.7) |
| CIDI-SF=Composite International Diagnostic Interview – short form | | | | | | | | | |

| **Table S14.** Ethnic difference in CIDI-SF scores and items among those who completed the MHQ follow-up in 2016 with a lifetime history of depression (N=48,464) | | | | | | | | | |
| --- | --- | --- | --- | --- | --- | --- | --- | --- | --- |
|  | CIDI-SF Depression | CIDI-SF core symptom: Prolonged sadness | CIDI-SF core symptom: Prolonged loss of interest | CIDI-SF non-core symptom:  Felt tired or had little energy | CIDI-SF non-core symptom:  Significant weight change | CIDI-SF non-core symptom:  Trouble sleeping (too much or too little) | CIDI-SF non-core symptom:  Trouble concentrating | CIDI-SF non-core symptom:  Feelings of worthlessness | CIDI-SF non-core symptom:  Thoughts of death |
|  | *OR (95% CI),*  *p value* | *OR (95% CI),*  *p value* | *OR (95% CI),*  *p value* | *OR (95% CI),*  *p value* | *OR (95% CI),*  *p value* | *OR (95% CI),*  *p value)* | *OR (95% CI),*  *p value* | *OR (95% CI),*  *p value* | *OR (95% CI),*  *p value* |
| White | Ref | Ref | Ref | Ref | Ref | Ref | Ref | Ref | Ref |
| Black | 0.86 (0.67 to 1.10), 0.226 | 0.54 (0.37 to 0.78), 0.001* | 0.72 (0.54 to 0.96), 0.025* | 0.46 (0.30 to 0.69), <0.001** | 1.52 (1.07 to 2.16), 0.018* | 1.15 (0.70 to 1.89), 0.579 | 0.53 (0.35 to 0.80), 0.002* | 0.55 (0.42 to 0.72), <0.001** | 0.88 (0.67 to 1.16), 0.371 |
| Mixed | 0.77 (0.61 to 0.97), 0.025* | 0.60 (0.42 to 0.86), 0.004* | 0.81 (0.62 to 1.07), 0.121 | 0.77 (0.49 to 1.22), 0.269 | 1.29 (0.96 to 1.74), 0.096 | 0.73 (0.49 to 1.07), 0.111 | 0.72 (0.47 to 1.10), 0.126 | 0.99 (0.75 to 1.31), 0.944 | 1.09 (0.84 to 1.42), 0.492 |
| Other | 0.83 (0.65 to 1.06), 0.131 | 0.69 (0.46 to 1.02), 0.067 | 0.93 (0.69 to 1.26), 0.650 | 0.69 (0.44 to 1.10), 0.122 | 1.20 (0.87 to 1.67), 0.260 | 1.07 (0.67 to 1.71), 0.783 | 0.62 (0.40 to 0.94), 0.024* | 0.92 (0.69 to 1.23), 0.577 | 0.90 (0.69 to 1.17), 0.435 |
| Other Asian | 0.98 (0.67 to 1.42), 0.906 | 0.81 (0.43 to 1.50), 0.485 | 0.77 (0.50 to 1.17), 0.226 | 0.65 (0.34 to 1.26), 0.202 | 0.82 (0.54 to 1.24), 0.354 | 1.66 (0.72 to 3.78), 0.231 | 0.63 (0.34 to 1.19), 0.154 | 0.82 (0.53 to 1.26), 0.356 | 1.22 (0.80 to 1.85), 0.353 |
| South Asian | 0.78 (0.61 to 1.01), 0.056 | 0.44 (0.31 to 0.61), <0.001** | 0.68 (0.52 to 0.90), 0.007* | 0.69 (0.43 to 1.09), 0.114 | 1.49 (1.07 to 2.07), 0.019* | 0.91 (0.59 to 1.42), 0.687 | 0.70 (0.44 to 1.10), 0.123 | 0.87 (0.65 to 1.17), 0.370 | 1.24 (0.92 to 1.65), 0.154 |
| Missing/unknown | 1.33 (0.95 to 1.86), 0.098 | 1.26 (0.68 to 2.32), 0.463 | 1.30 (0.86 to 1.97), 0.212 | 1.77 (0.78 to 4.03), 0.170 | 1.06 (0.72 to 1.56), 0.765 | 0.81 (0.48 to 1.37), 0.438 | 1.23 (0.62 to 2.42), 0.553 | 1.40 (0.94 to 2.09), 0.100 | 1.26 (0.88 to 1.80), 0.200 |
| *p<0.05; **p<0.001  Fully adjusted analyses: Age, sex, Townsend deprivation quintile, number of physical long-term conditions  CI=confidence interval; CIDI-SF=Composite International Diagnostic Interview – short form; OR=odds ratio  Note: Benjamini–Hochberg false discovery rate (5%) applied as a sensitivity analysis; contrasts with p‑values below 0.010 were considered significant after adjustment. | | | | | | | | | |

| **Table S15.** Ethnic difference in the likelihood of missingness for each CIDI-SF item among those who completed the MHQ follow-up in 2016 with a lifetime history of depression (N=48,464) | | | | | | | | |
| --- | --- | --- | --- | --- | --- | --- | --- | --- |
|  | CIDI-SF core symptom: Prolonged sadness | CIDI-SF core symptom: Prolonged loss of interest | CIDI-SF non-core symptom:  Felt tired or had little energy | CIDI-SF non-core symptom:  Significant weight change | CIDI-SF non-core symptom:  Trouble sleeping (too much or too little) | CIDI-SF non-core symptom:  Trouble concentrating | CIDI-SF non-core symptom:  Feelings of worthlessness | CIDI-SF non-core symptom:  Thoughts of death |
|  | *OR (95% CI),*  *p value* | *OR (95% CI),*  *p value* | *OR (95% CI),*  *p value* | *OR (95% CI),*  *p value* | *OR (95% CI),*  *p value* | *OR (95% CI),*  *p value* | *OR (95% CI),*  *p value* | *OR (95% CI),*  *p value* |
| White | Ref | Ref | Ref | Ref | Ref | Ref | Ref | Ref |
| Black | 3.79 (0.50 to 28.43), 0.196 | 2.58 (0.35 to 18.93), 0.352 | 1.32 (0.92 to 1.89), 0.128 | 1.17 (0.86 to 1.58), 0.320 | 0.98 (0.68 to 1.41), 0.918 | 1.21 (0.84 to 1.72), 0.306 | 1.35 (0.95 to 1.92), 0.098 | 1.30 (0.92 to 1.84), 0.142 |
| Mixed | 5.84 (1.39 to 24.51), 0.016* | 3.90 (0.95 to 16.06), 0.059 | 1.59 (1.17 to 2.16), 0.003* | 1.15 (0.87 to 1.52), 0.312 | 1.45 (1.09 to 1.94), 0.011* | 1.39 (1.02 to 1.90), 0.035* | 1.48 (1.08 to 2.02), 0.015* | 1.37 (1.00 to 1.87), 0.052 |
| Other | - | - | 1.28 (0.90 to 1.82), 0.162 | 1.31 (0.99 to 1.75), 0.063 | 1.04 (0.74 to 1.46), 0.838 | 1.42 (1.03 to 1.97), 0.034* | 1.37 (0.98 to 1.93), 0.066 | 1.01 (0.69 to 1.47), 0.965 |
| Other Asian | 7.94 (1.08 to 58.31), 0.042* | - | 0.90 (0.49 to 1.64), 0.732 | 0.69 (0.41 to 1.17), 0.166 | 0.63 (0.34 to 1.18), 0.152 | 1.14 (0.67 to 1.93), 0.630 | 1.52 (0.93 to 2.49), 0.094 | 0.95 (0.53 to 1.68), 0.850 |
| South Asian | - | 2.36 (0.33 to 17.08), 0.395 | 1.50 (1.07 to 2.09), 0.018* | 1.16 (0.86 to 1.56), 0.333 | 1.14 (0.82 to 1.60), 0.439 | 1.60 (1.16 to 2.19), 0.004* | 1.52 (1.09 to 2.12), 0.013* | 1.57 (1.13 to 2.16), 0.006* |
| Missing/unknown | - | - | 0.59 (0.32 to 1.06), 0.076 | 1.05 (0.72 to 1.55), 0.791 | 0.94 (0.60 to 1.47), 0.793 | 0.90 (0.55 to 1.45), 0.661 | 0.96 (0.59 to 1.55), 0.872 | 0.79 (0.47 to 1.33), 0.378 |
| *p<0.05; **p<0.001  Fully adjusted analyses: Age, sex, Townsend deprivation quintile, number of physical long-term conditions  CI=confidence interval; CIDI-SF=Composite International Diagnostic Interview – short form; OR=odds ratio  Note: Benjamini–Hochberg false discovery rate (5%) applied; no contrasts remained significant after adjustment. | | | | | | | | |


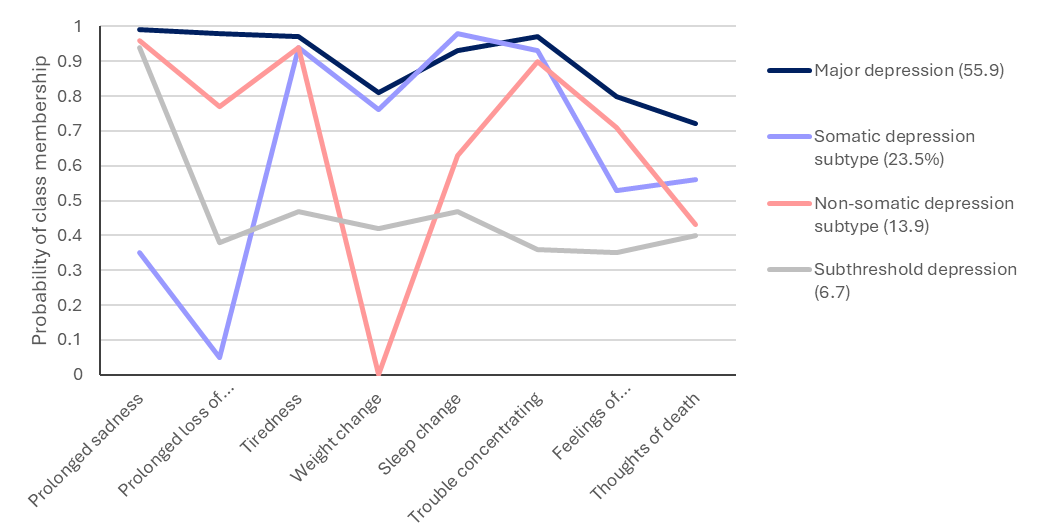


**Figure S1.** Four depression profiles (latent classes) which emerged from the latent class analysis based on CIDI-SF item endorsement in those with a lifetime history of depression who completed the 2022 Mental Health Questionnaire follow-up (N=51,721). The probabilities of class membership represent the model-estimated likelihood (ranging from 0 to 1) that participants within each latent class endorse a given symptom.

|  | White | Black | Mixed | Other | Other Asian | South Asian | Missing/unknown |
| --- | --- | --- | --- | --- | --- | --- | --- |
| Prolonged sadness | 91.9 | 88.7* | 88.8* | 89.8 | 91.0 | 84.0** | 93.3 |
| Prolonged loss of interest | 79.3 | 79.2* | 78.7 | 80.3 | 77.2 | 75.0* | 83.6 |
| Tiredness | 93.5 | 89.2** | 92.7 | 91.6 | 91.0 | 91.2 | 96.1 |
| Weight change | 69.9 | 82.8* | 77.8 | 76.5 | 66.4 | 77.5* | 70.4 |
| Sleep change | 90.9 | 93.2 | 89.1 | 91.9 | 94.6 | 90.3 | 88.7 |
| Trouble concentrating | 92.6 | 89.2* | 91.3 | 89.1* | 89.7 | 90.5 | 93.8 |
| Feelings of worthlessness | 71.7 | 66.0** | 76.2 | 72.6 | 70.2 | 72.7 | 78.8 |
| Thoughts of death | 66.0 | 67.6 | 70.1 | 65.8 | 71.8 | 71.4 | 71.1 |

**Figure S2.** Heat map showing the percentage frequency of depression symptoms (CIDI-SF) reported during the 2016 mental health questionnaire (MHQ) follow-up among individuals with a lifetime history of depression (N = 48,464). Colour intensity ranges from green to red, with red indicating higher symptom frequency. Significant differences detected using logistic regression models are indicated using asterisks.*p<0.05,**p<0.001
